# Impact of Condensed Tannin and Sulfur Dioxide Addition on Acetaldehyde Accumulation and Anthocyanin Profile of *Vitis vinifera* L. Cv. Cabernet Sauvignon Wines During Alcoholic Fermentation

**DOI:** 10.3390/molecules29225238

**Published:** 2024-11-05

**Authors:** Qinglong Wang, Xiaoqian Cui, Jiaqi Wang, Heqiang Chang, Junzhe Wang, Ang Zhang, Yang Zhou, Zhiyong Xu, Lingmin Dai, Guomin Han

**Affiliations:** 1 School of Bioengineering, Qilu University of Technology (Shandong Academy of Sciences), Jinan 250353, China; wangqinglong975@163.com (Q.W.); 17863908085@163.com (X.C.); 19861406968@163.com (J.W.); 19862126289@163.com (H.C.); wjz001118@163.com (J.W.); zhywine@163.com (Y.Z.); xzy19890710@126.com (Z.X.); 2 Technology Center of Qinhuangdao Customs, Qinhuangdao 066004, China; zhanganggrape@hotmail.com; 3 Wei Long Grape Wine Co., Ltd., Yantai 265704, China

**Keywords:** wine, acetaldehyde, anthocyanin, Malvidin-3-O-glucoside, alcoholic fermentation

## Abstract

Acetaldehyde is a key carbonyl by-product during red wine alcoholic fermentation; it is reactive and takes part in certain reactions involving anthocyanin. The aim of this study was to investigate the influence of SO_2_ and condensed tannin on the acetaldehyde accumulation of *Saccharomyces cerevisiae* (*S. cerevisiae*) during alcoholic fermentation and the ripple effect on wine anthocyanin. In this study, six sets of Cabernet Sauvignon alcoholic fermentation with two different sulfur levels (HS and LS) were carried out by adding exogenous condensed tannins before fermentation (T0) in the acetaldehyde rise period (TA) of *S. cerevisiae* and at the end of fermentation (TE), separately. The acetaldehyde evolution was identified during fermentation and anthocyanin was analyzed comparatively. The results showed that HS treatment slowed down the degradation of acetaldehyde, while tannins accelerated the degradation of acetaldehyde during alcoholic fermentation, especially TA wines. Furthermore, TA wines possessed a unique anthocyanin profile after fermentation regardless of SO_2_ level compared with other wines. These results suggest that acetaldehyde-mediated anthocyanin polymerization most likely occurs timely at the acetaldehyde production phase of *S. cerevisiae* during alcoholic fermentation, and managing tannin addition time during production could be used to regulate the anthocyanin profile.

## 1. Introduction

The anthocyanin profile determines the color of red wine, which is an essential factor for consumers in determining a purchase and indicates the wine’s quality and stability [1]. Its characteristic depends on the anthocyanins extracted from the grape skin during the maceration process, as well as the stable derived pigment formation from complex reactions during fermentation and aging [2]. During the vinification, some other compounds in wine lead to the reduction of monomer anthocyanins and form more stable anthocyanin-derived polymer pigments [3]. Among them, pyranoanthocyanins mainly generate in the process of fermentation and aging, that is, monomer anthocyanins and acylated anthocyanins react with acetaldehyde, pyruvate, vinylphenol, α-ketoglutaric acid, and other compounds to form cycloaddition compounds [4]. Direct condensation between anthocyanins and falvan-3-ols occurs through a linkage between the electrophilic flavylium form of an anthocyanin unit and the nucleophilic A-ring of a catechin unit (a tannin subunit), and the A-ring on anthocyanins and catechins can also generate wine pigments via condensation with acetaldehyde [5]. Research has demonstrated that acetaldehyde is crucial in creating polymeric pigments [6], and the ethyl bridging tannin–anthocyanin condensation rate is 120 times quicker than direct covalent bonding [7].

Appropriate yeast inoculation can effectively boost the synthesis of anthocyanin derivatives by metabolizing more pyruvate and acetaldehyde [8]. *S. cerevisiae* generates acetaldehyde as a by-product in glycolysis by converting pyruvate during fermentation, and there are many factors affecting acetaldehyde metabolism in wine, including yeast strain and fermentation conditions, such as SO_2_ levels, pH, and temperature [9]. Due to several factors, acetaldehyde levels in red wine vary from 4 to 212 mg/L [10], and this variation can significantly affect the process of acetaldehyde-induced pigment polymerization, speeding up ethyl-bridging pigment formation and improving the color of the wine [11]. Recent studies demonstrated that acetaldehyde levels during alcohol fermentation impress the synthesis of pyranoid anthocyanins and polymeric anthocyanins [12,13].

As the frequently utilized antioxidant in vinification, the presence of SO_2_ may also enhance the concentration of acetaldehyde during wine fermentation [14] due to the harmful impact of free SO_2_ on yeast, which promotes acetaldehyde generation to bind with free SO_2_ to create a detoxifying mechanism, while acetaldehyde bound to SO_2_ could not be metabolized by the yeast [15]. It was demonstrated that SO_2_ addition both affects yeast and non-yeast metabolisms, resulting in an average acetaldehyde synthesis of 325 µg per milligram of SO_2_ addition [10]. In the early stages of co-fermentation, bacterial survival was closely related with the early, transient formation of high acetaldehyde production from specific SO_2_-producing yeast strains [16]. However, the amount of SO_2_ added is limited due to its allergic reactions, and it is necessary to maximize the effect of the limited content of acetaldehyde as much as possible.

Enological tannins are always used as allowed substitutes for SO_2_ in order to control oxidization during vinification [17], the oxygen consumption rate was confirmed to follow second-order kinetics in a model wine solution that contained different oenological tannins [18]. Otherwise, tannins also participate in chemical reactions during winemaking processes, resulting in variation in color through condensation derivatives with anthocyanins, which is beneficial to the color improvement and stabilization of red wines [19,20], and the reaction always involves the participation of acetaldehyde [21]. However, whether the tannin addition stage regulates acetaldehyde accumulation during alcohol fermentation has not been indicated, and its impact on anthocyanin profile is also deserving of discussion. This present study aimed to assess the effect of SO_2_ and tannin supplementation on the accumulation of acetaldehyde during alcohol fermentation, as well as the relationship between the accumulation characteristics of acetaldehyde and the variation of anthocyanin.

## 2. Results and Discussion

### 2.1. Acetaldehyde Accumulation During the Fermentation

**Figure 1:**
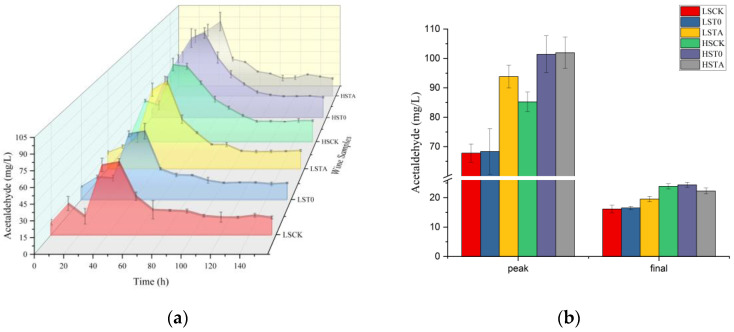
Variation tendency of acetaldehyde (**a**) and peak/final acetaldehyde (**b**) during fermentation. Abbreviations: LSCK, control wine with low sulfur; LST0, wine with low sulfur and addition of exogenous condensed tannins before fermentation; LSTA, wine with low sulfur and addition of exogenous condensed tannins in the rise period of acetaldehyde; HSCK, control wine with high sulfur; HST0, wine with high sulfur and addition of exogenous condensed tannins before fermentation; HSTA, wine with high sulfur and addition of exogenous condensed tannins in the rise period of acetaldehyde.

The acetaldehyde evolution of different treatment groups during vinification is shown in Figure 1a; the TE wines were not shown as they were similar to the corresponding control wines. All the wines displayed similar accumulation kinetics where acetaldehyde reached an initial peak value within 40 h after the beginning of fermentation followed by partial reutilization, this observation is consistent with the data published in previous studies [10,15]. However, with different SO_2_ treatment, the HS group always contained significantly higher acetaldehyde peak values than the counterpart of the LS group; furthermore, the final acetaldehyde value after fermentation in all the HS groups was dramatically higher than all the LS groups, as shown in Figure 1b. Earlier research demonstrated that the production of early acetaldehyde correlates with the duration of the lag phase and that the excretion of this early acetaldehyde may be related to the detoxification of SO_2_ [22], and the peak value of free acetaldehyde was found when SO_2_ concentration equilibrium between diffusion and production was achieved [15]. In this way, the accumulation of acetaldehyde is usually considered an early marker of yeast fermentation activity [15]. Actually, sulfur addition always led to a slow accumulation of acetaldehyde during alcoholic fermentation over time, even at the end of fermentation, regardless of cooling or the apparent inhibition of yeast sugar metabolism [23].

As shown in Figure 1, it is interesting to note that the addition of exogenous tannins also played a certain role in acetaldehyde accumulation. The peak values of acetaldehyde during the experiment in the samples with different SO_2_ levels were slightly different, both acetaldehyde peak values of T0 and TA wines in HS groups were higher than CK, while only TA wines in LS groups were higher than CK. The final value of LSTA was also the highest among the LS groups, like peak value, but the HS groups exhibited different results. Therefore, it seems that the production and consumption of acetaldehyde are not only related to SO_2_ but also strongly associated with the addition of tannins. As a key parameter for monitoring wine non-enzymatic oxidation, acetaldehyde generated from a well-known Fenton reaction was always monitored [24,25,26]. The less production of acetaldehyde during oxidation was verified when higher exogenous oligomeric tannins were added to red wine [27]; on the contrary, more acetaldehyde generation was found during oxidation when three different tannins, gallotannins, ellagitannins, and condensed tannins, were added to red wine, and then the generated acetaldehyde was consumed in 30 days [28]. However, no study has been undertaken to investigate the detailed mechanism of the influence of different addition times of enological tannin on acetaldehyde variation induced by yeast metabolism during alcoholic fermentation.

**Figure 2:**
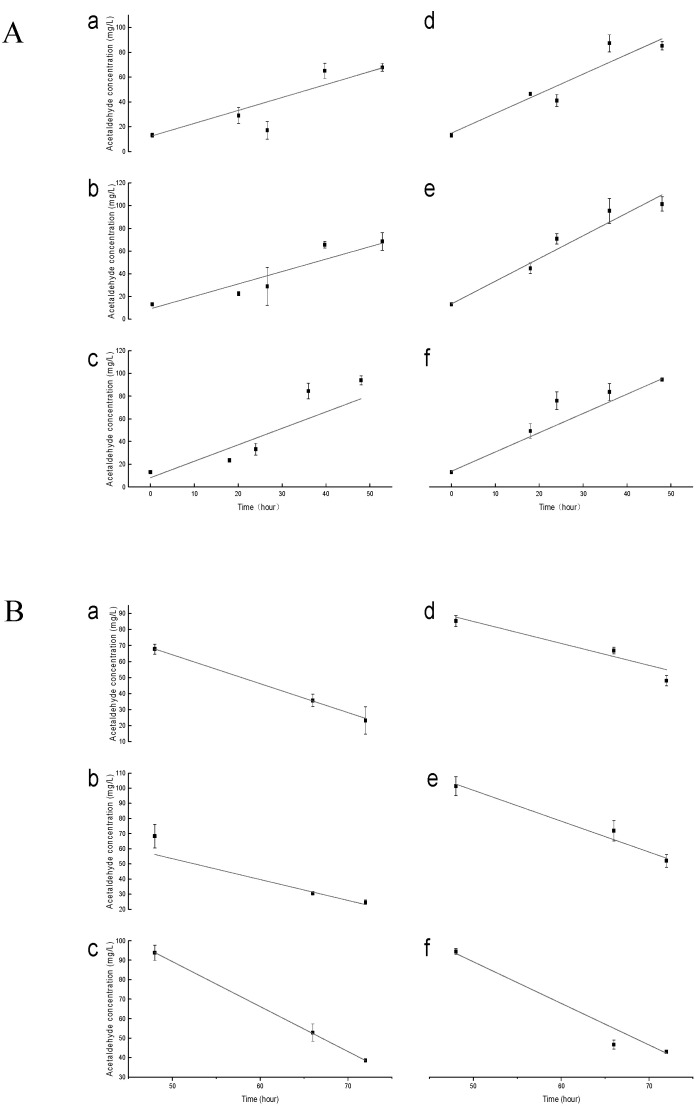
The peak production rate (**A**) and consumption rate (**B**) of acetaldehyde during fermentation were linearly fitted. Note: **a** indicates LSCK processing; **b** indicates LSTO processing; **c** indicates LSTA processing; **d** indicates HSCK processing; **e** indicates HSTO processing; **f** indicates HSTA processing. The TE wines were not shown as they were similar to CK wines. Abbreviation: LSCK, control wine with low sulfur; LST0, wine with low sulfur and addition of exogenous condensed tannins before fermentation; LSTA, wine with low sulfur and addition of exogenous condensed tannins in the rise period of acetaldehyde; HSCK, control wine with high sulfur; HST0, wine with high sulfur and addition of exogenous condensed tannins before fermentation; HSTA, wine with high sulfur and addition of exogenous condensed tannins in the rise period of acetaldehyde.

After the linear fitting of the peak formation rate and degradation rate of acetaldehyde (Table 1 and Figure 2), it was found that the formation rate of acetaldehyde could be promoted, and the degradation rate of acetaldehyde could be slowed down under the action of a high concentration of SO_2_. Different concentrations of SO_2_ had an impact on the maximum content of acetaldehyde: at high concentrations, the accumulation rate of acetaldehyde was 1.39 times that of the LS group, and the maximum acetaldehyde content was 1.25 times that of the LS group.

When the acetaldehyde content began to decline, the acetaldehyde degradation rate of the HS group was 0.76 times that of the LS group. At the same time, the bound SO_2_ content remained at 12.7 mg/L and 31.7 mg /L, respectively (Table 2). The results indicated that the content of SO_2_ hindered the degradation rate of acetaldehyde at a high level compared with the sulfur content at a low level. The overproduction of acetaldehyde may be due to the inhibition of ethanol dehydrogenase, which prevents acetaldehyde from converting to ethanol, and the binding of acetaldehyde to SO_2_, which results in a decrease in the amount metabolized to ethanol [15].

In the LS group, although T0 had no significant influence on the peak production rate and concentration of acetaldehyde, it was 0.24 times slower than CK when acetaldehyde reached its peak consumption, which seemed to slow down the breakdown rate of acetaldehyde. However, LSTA can accelerate the consumption of acetaldehyde. The degradation rate of acetaldehyde is 1.28 times that of CK, and the maximum concentration of acetaldehyde is 1.38 times that of CK. These data indicate that when the initial total sulfur content is modest (around 20 mg/L), adding exogenous tannins when acetaldehyde reaches its peak can accelerate acetaldehyde reduction. When there is a specific concentration of acetaldehyde in the fermentation mash, the addition of exogenous tannins is a consumer substance for acetaldehyde, and the rate of acetaldehyde-mediated tannin–anthocyanin condensation is fast [29], which may be the reason why the addition of tannins during the stage of the acetaldehyde production accelerates the degradation rate of acetaldehyde.

Similarly, adding tannins can improve the rate of acetaldehyde metabolism in the HS group. Unlike the LS group, the effect of HST0 on acetaldehyde metabolism was positive. Interestingly, HS content will inhibit the rate of acetaldehyde content falling, but the superposition of tannins can increase the decomposition rate of acetaldehyde. The drop in acetaldehyde concentration during the fixation phase is primarily due to yeast consumption, which the metabolic relationship between acetaldehyde and metabolic indicators can explain [8]. This study has demonstrated once again that the addition of tannins can consume acetaldehyde during alcohol fermentation, indicating that acetaldehyde-mediated polymerization occurs during the short peak period of acetaldehyde. However, with the addition of exogenous tannins, the metabolism rate of acetaldehyde in the HS group was faster than in the LS group. The concentration of acetaldehyde in the HS group was generally higher than in the LS group. In addition to the consumption of acetaldehyde by yeast, the added exogenous tannins in the TA wines also easily reacted with it.

### 2.2. Anthocyanin Analysis

**Figure 3:**
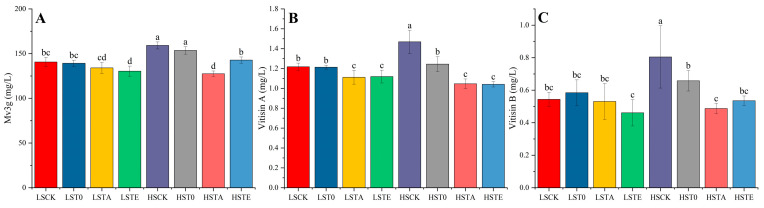
The concentration of Mv3g (**A**) and pyranoid anthocyanins (**B**,**C**) in different wines after 30 days aging. Abbreviation: Mv3g: Malvidin 3-O-glucoside; LSCK, control wine with low sulfur; LST0, wine with low sulfur and addition of exogenous condensed tannins before fermentation; LSTA, wine with low sulfur and addition of exogenous condensed tannins in the rise period of acetaldehyde; LSTE, wine with low sulfur and addition of exogenous condensed tannins at the end of fermentation; HSCK, control wine with high sulfur; HST0, wine with high sulfur and addition of exogenous condensed tannins before fermentation; HSTA, wine with high sulfur and addition of exogenous condensed tannins in the rise period of acetaldehyde; HSTE, wine with high sulfur and addition of exogenous condensed tannins at the end of fermentation. The HPLC chromatography can be found in Figure S1. Note: the lowercase letters a, b, c, and d indicate a significant difference (*p* < 0.05).

Anthocyanin is the origin of the intense red color observed in red wines, especially young red wines with Malvidin-3-O-glucoside (Mv3g) being the most abundant compound [30]. Thus, this compound and its derivative were required primarily in this research. The differences in Mv3g among free-run wine samples after 30 days of aging are shown in Figure 3A, which ranged from 125 to 160 mg/L. Due to the protective effect of SO_2_, HS wines should contain a higher level of Mv3g compared with LS wines, such as HSCK and HST0. However, HSTA wines contained the lowest level of Mv3g, which was significantly lower than the other three HS groups and LSCK/LST0 wines (*p* < 0.05). This result illustrated that tannin addition in the acetaldehyde production period tempestuously accelerated the chemical reaction of Mv3g. As we know, acetaldehyde produced from yeast metabolism can directly react with Malvidin-3-glucoside to produce Vitisin B [31], or participate in the formation of ethyl-bridged anthocyanins and flavonols [32]. As Figure 1b shows, both HST0 and HSTA endured the highest acetaldehyde peak (Figure 1b), but only HSTA wines were shown to have obviously different levels of Mv3g compared with CK and T0 wines within the group with the same level of SO_2_ (*p* < 0.05). It is interesting that Vitisin B in HSTA wines was not conspicuously higher than other groups, which could be concluded as the least stable pigments due to their chemical properties, as Vitisin B is only partly resistant to SO_2_ [33] and is reported to be less stable than anthocyanin (simulated solution with pH 1.5 and 7.5, respectively) [34]. It is noteworthy that TE wines also possessed obviously lower levels of Mv3g compared with CK and T0 wines within the group with the same level of SO_2_ (*p* < 0.05), while there were no significant differences between CK and T0 wines. These results demonstrate that the earlier addition of tannin before the acetaldehyde production period of yeast would not promote the evolution of Mv3g.

**Figure 4:**
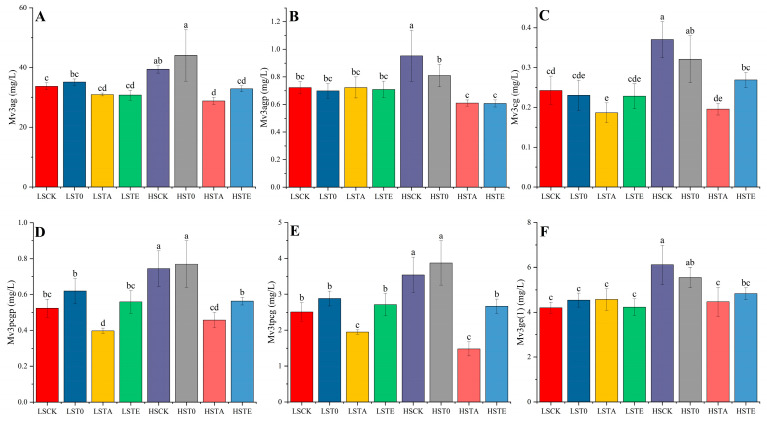
The concentration of acylated anthocyanins (**A**–**E**) and polymeric anthocyanins (**F**) in different wines after 30 days aging. Abbreviation: Mv3ag: Malvidin 3-O-(6-O-acetyl)-glucoside; Mv3agp: Malvidin 3-O-(6-O-acetyl)-glucoside-pyruvic acid; Mv3cg: Malvidin 3-O-(6-O-caffeoyl)-glucoside; Mv3pcgp: Malvidin 3-O-(6-O-p-coumaryl)-glucoside-pyruvic acid; Mv3tpcg: Malvidin 3-O-(6-O-trans-p-coumaryl)-glucoside; Mv3ge (1): Malvidin 3-O-glucoside-ethyl-catechin (1); LSCK, control wine with low sulfur; LST0, wine with low sulfur and addition of exogenous condensed tannins before fermentation; LSTA, wine with low sulfur and addition of exogenous condensed tannins in the rise period of acetaldehyde; LSTE, wine with low sulfur and addition of exogenous condensed tannins at the end of fermentation; HSCK, control wine with high sulfur; HST0, wine with high sulfur and addition of exogenous condensed tannins before fermentation; HSTA, wine with high sulfur and addition of exogenous condensed tannins in the rise period of acetaldehyde; HSTE, wine with high sulfur and addition of exogenous condensed tannins at the end of fermentation. The HPLC chromatography can be found in Figure S1. Note: the lowercase letters a, b, c, d, and e indicate a significant difference (*p* < 0.05).

The alterations in acylated anthocyanins are displayed in Figure 4A–E for Mv3ag, Mv3agp, Mv3cg, Mv3pcgp, and Mv3tpcg, respectively. The results show that Mv3ag and Mv3agp in the LS groups had insignificant differences (*p* > 0.05), while HSCK and HST0 contained the highest number of compounds HSTA contained the lowest number of compounds, as shown for Mv3G. Nevertheless, LSTA wines contained significantly lower levels of Mv3pcgp and Mv3tpcg compared with other wines, except for the HSTA wine. For Mv3tpcg, the level of this compound in HSCK and HST0 wines was significantly higher than in the other wines.

**Figure 5:**
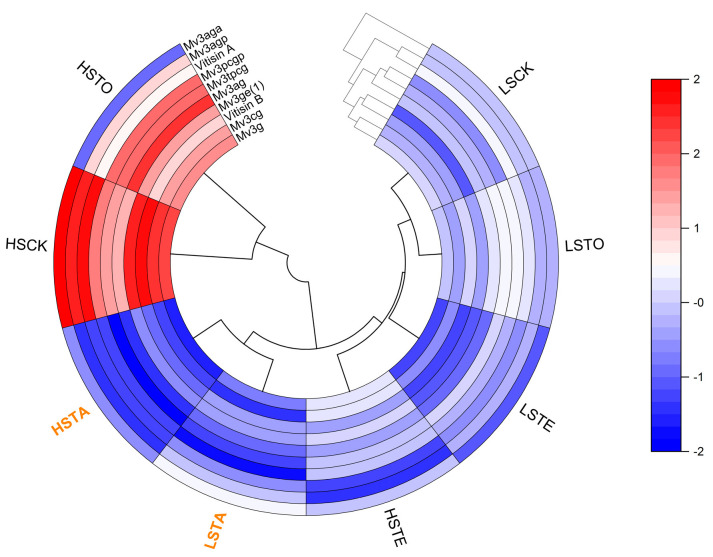
Complete linkage hierarchical clustering (dendrogram) for 10 wine samples with different treatments through the measure of Euclidean distances, using anthocyanins data after 30 days aging. Abbreviation: Mv3aga: Malvidin 3-O-(6-O-acetyl)-glucoside-acetaldehyde; Mv3ag: Malvidin 3-O-(6-O-acetyl)-glucoside; Mv3agp: Malvidin 3-O-(6-O-acetyl)-glucoside-pyruvic acid; Mv3cg: Malvidin 3-O-(6-O-caffeoyl)-glucoside; Mv3pcgp: Malvidin 3-O-(6-O-p-coumaryl)-glucoside-pyruvic acid; Mv3tpcg: Malvidin 3-O-(6-O-trans-p-coumaryl)-glucoside; Mv3ge (1): Malvidin 3-O-glucoside-ethyl-catechin (1); Mv3g: Malvidin 3-O glucoside; LSCK, control wine with low sulfur; LST0, wine with low sulfur and addition of exogenous condensed tannins before fermentation; LSTA, wine with low sulfur and addition of exogenous condensed tannins in the rise period of acetaldehyde; LSTE, wine with low sulfur and addition of exogenous condensed tannins at the end of fermentation; HSCK, control wine with high sulfur; HST0, wine with high sulfur and addition of exogenous condensed tannins before fermentation; HSTA, wine with high sulfur and addition of exogenous condensed tannins in the rise period of acetaldehyde; HSTE, wine with high sulfur and addition of exogenous condensed tannins at the end of fermentation.

To organize these obtained anthocyanin values into meaningful structures, the selected ten anthocyanin variables were illustrated by the tree of hierarchical clustering of the Euclidean distances (dendrogram), as shown in Figure 5. Because all wine samples showed no significant difference in the concentration of Mv3aga, it was not exhibited in Figure 3 and Figure 4. The complete linkage of hierarchical clustering clearly differentiates between HSCK/HST0 red wines and the other wine samples, and this means that the protective effect of sulfur in HSCK and HST0 wines was obvious but the tannin addition weakened its effect in the other HS wines. Within the main cluster of the other wines, the samples are correctly assigned to subclusters as TA and LS/TE wines, where a distinct discrepancy could be observed, further confirming the crucial influence of tannin addition in the acetaldehyde rise period on anthocyanin. These clustering results were also in accordance with the anthocyanin variation among these eight wine samples shown in Figure 3 and Figure 4.

## 3. Materials and Methods

### 3.1. Reagents and Standards

Condensed tannin was provided by Laffort (Floirac, France). Potassium metabisulfite was purchased from Enartis Winy (Novara, Italy). Acetonitrile, methanol, and formic acid were purchased from Merck (HPLC grade, Darmstadt, Germany). The HPLC standard solution of acetaldehyde-2, 4-dinitrophenyrazine (DNPH) was purchased from the Beijing North Weiye Institute of Metrology Technology (Beijing, China). Sodium hydroxide, hydrogen peroxide, and sulfuric acid were purchased from Sinopharm Chemical Reagent Co., Ltd. (Shanghai, China); phosphoric acid was purchased from Tianjin Kemiou Chemical Reagent Co., Ltd. (Tianjin, China).

### 3.2. Process of Vinification

The 2022 Cabernet Sauvignon grape from Ningxia, China, was hand-selected for destemming and pressing. The grape must was then placed into twelve 20 L fermentation vats, with vats 1–6 receiving 20 ppm SO_2_ (measured as 200% potassium sulfite) and vats 6–12 receiving 60 ppm SO_2_. The samples were incubated with a Brix degree of 23.5°, a pH of 3.4, and a titratable acid of 6.5 g/L. Dry yeast powder (FX10 from Lafford, France) was inoculated at a rate of 25 g/hL and incubated in warm water at 40 °C for 20 min. When the temperature differential between the grape juice and the yeast was less than 10 °C, yeast was added. Treatments during fermentation consisted of different sulfur concentrations, 20 ppm SO_2_ (LS) and 60 ppm SO_2_ (HS), and the addition of 100 mg/L exogenous tannins at various stages of fermentation: control without addition (CK), before fermentation (T0), and in the acetaldehyde rise period (TA) and at the end of fermentation (TE). All the experimental wines were prepared in duplicate. Every twelve hours, the temperature, acetaldehyde levels, and specific gravity were recorded. After fermentation, free-run wine was collected and stored in 5 L sealed glass jar. The physicochemical indexes and anthocyanin levels were determined after 30 days of aging. The physicochemical indexes are listed in Table 3, regarding the International Vine and Wine Organization (OIV) method for measuring pH, sulfur dioxide, residual sugar, total acid, volatile acid, and other conventional physicochemical indices.

### 3.3. Acetaldehyde Determination

The derivatization of the DNPH-LC method was selected to analyze acetaldehyde in the wines [35]. In a certain sequence, 20 μL of 25% sulfuric acid, 20 μL of potassium metabisulfite solution (2.24 g/L), and 240 μL of DNPH (6 g/L) were added to 120 μL wine samples. The reaction was in a water bath at 65 °C for 15 min, and after cooling to room temperature, the solution was diluted 1:1 with 35% acetonitrile ultrapure water and mixed thoroughly. The reaction solution that had been thoroughly derivatized was filtered using a 0.22 μm PTFE membrane filter and subjected to analysis using a 2998 diode array detector in Waters e2695 high-performance liquid chromatography (Milford, MA, USA) at 365 nm, and the chromatographic column was an Agilent ZORBAX fast resolution HT, SB-C18 (1.8 μm, 4.6 × 100 mm, Palo Alto, CA, USA) at 35 °C with a flow rate of 0.75 mL/min. The external standard method was applied for the quantitative analysis, and linear regression analysis was performed. Acetaldehyde–DNPH was identified by comparison of retention times and chromatograms with those reported by Han et al. [35]. Data analysis and peak integration were performed using an Empower 3.6.0 chromatographic workstation.

### 3.4. Anthocyanin Determination

Anthocyanins were analyzed according to a published method [34]. Wine samples were directly injected into a LC (Shimadzu LC-20A, Shimadzu, Kyoto, Japan) equipped with a Synergi Hydro-RP C18 column (250 × 4.6 mm, 4 μm, Phenomenex, Torrance, CA, USA) at 520 nm, after the samples were filtered through a 0.22 μm water filter. UV detection was obtained using a diode array detector (DAD). The chromatographic conditions were as follows: column temperature 35 °C; injection volume 20 μL; flow rate 1.000 mL/min; mobile phase A: 2.5% formic acid in water: acetonitrile = 8:1 (*v*/*v*); mobile phase B: 2.5% formic acid in water: acetonitrile = 4:5 (*v*/*v*); elution gradient: 0~45 min, 0% B~35% B; 45~46 min, 35% B~100% B; 46~50 min, 100% B~100% B; 50~51 min, 100% B~0% B; 51~55 min, 0% B~0% B. The identification of the observed anthocyanins was based on their UV–Vis spectra, and their retention time was compared with a published method in which anthocyanins were identified using UPLC-ESI-MS/MS [34]. The relative concentration of each anthocyanin was calculated using the external traditional method and quantified using Malvidin 3-O-glucoside.

### 3.5. Data Analysis

Data were expressed as the mean of four values (2 experimental replicates × 2 analytical replicates) and reported along with their standard deviation (S.D). Regression analysis and one-way analysis of variance (ANOVA) (*p* < 0.05) were performed using the IBM SPSS Statistics 25 (IBM Corp., Armonk, NY, USA). Differences among wine samples (Figure 2, Figure 3 and Figure 4) were determined by Fisher’s Least Significant Difference. Cluster analysis was performed using Originpro 2024b software with the Polar Heatmap-app v1.10 (OriginLab Corp., Northampton, MA, USA) to obtain biplot graphics (Figure 5).

## 4. Conclusions

This study demonstrates the influence of sulfur dioxide levels and tannin addition stages on acetaldehyde variation during alcoholic fermentation. The acetaldehyde accumulation content in the HS group was higher during the acetaldehyde production phase, but tannin addition at different stages may weaken its effect on acetaldehyde accumulation of *S. cerevisiae*, especially in TA wines regardless of sulfur level.

The high level of SO_2_ slowed down the degradation rate of acetaldehyde during its consumption period due to its binding action. In contrast, the addition of tannins accelerated the degradation rate, especially when the tannin was added at the phase of acetaldehyde production, under the circumstances, acetaldehyde maybe consumed by chemical reactions quickly, which convert monomer anthocyanins into macromolecular anthocyanins. These results illustrated that tannin addition at different periods of yeast acetaldehyde metabolism could cause different distinguished anthocyanin profiles, especially addition during the acetaldehyde production period.

Future studies could address the relationship between different types of exogenous tannin addition at different phases during wine fermentation, and its long-term effect on wine color during wine aging also should be concerned.

## Figures and Tables

**Table 1:** The slope, R-square, and peak value of acetaldehyde peak production rate and degradation rate after linear fitting.

Sample	Acetaldehyde Peak Formation		Acetaldehyde Peak Degradation	
	Slope	R2	Slope	R2
LSCK	1.140	0.952	−1.805	0.999
LST0	1.193	0.820	−1.380	0.883
LSTA	1.449	0.823	−2.311	1.000
HSCK	1.586	0.965	−1.368	0.870
HST0	2.001	0.979	−2.040	0.977
HSTA	1.697	0.993	−2.137	0.983

Abbreviation: LSCK, control wine with low sulfur; LST0, wine with low sulfur and addition of exogenous condensed tannins before fermentation; LSTA, wine with low sulfur and addition of exogenous condensed tannins in the rise period of acetaldehyde; HSCK, control wine with high sulfur; HST0, wine with high sulfur and addition of exogenous condensed tannins before fermentation; HSTA, wine with high sulfur and addition of exogenous condensed tannins in the rise period of acetaldehyde. Note: the TE wines were not shown as they were similar to CK wines.

**Table 2:** Content of bound SO_2_ during fermentation (mg/L).

Wine Sample	Time (Hour)				
	0	15	39	51	147
LSCK	10.73 ± 0.92	12.00 ± 2.36	23.60 ± 14.66	12.70 ± 3.52	0.00 ± 0.00
LST0	10.80 ± 0.41	7.40 ± 3.83	16.97 ± 3.24	16.00 ± 2.59	0.00 ± 0.00
LSTA	11.20 ± 0.40	0.80 ± 1.60	29.40 ± 4.10	23.50 ± 5.26	0.00 ± 0.00
HSCK	31.00 ± 0.72	48.40 ± 13.39	35.30 ± 18.59	31.70 ± 20.51	6.70 ± 3.05
HST0	31.13 ± 1.10	37.30 ± 10.56	55.50 ± 5.50	59.60 ± 5.76	7.30 ± 4.46
HSTA	32.40 ± 0.53	34.89 ± 5.73	54.00 ± 3.22	40.70 ± 1.15	7.10 ± 1.15

Abbreviation: LSCK, control wine with low sulfur; LST0, wine with low sulfur and addition of exogenous condensed tannins before fermentation; LSTA, wine with low sulfur and addition of exogenous condensed tannins in the rise period of acetaldehyde; HSCK, control wine with high sulfur; HST0, wine with high sulfur and addition of exogenous condensed tannins before fermentation; HSTA, wine with high sulfur and addition of exogenous condensed tannins in the rise period of acetaldehyde. Note: the TE wines were not shown as they were similar to CK wines.

**Table 3:** Physicochemical indexes of wine samples.

Wine Sample	pH	Total Acid (g/L)	Volatile Acid (mg/L)	Alcoholic Strength (%vol)	Residual Sugar (g/L)
LSCK	3.69 ± 0.02	8.56 ± 0.47	0.32 ± 0.045	13.1 ± 0.1	2.28 ± 0.63
LST0	3.67 ± 0.02	8.26 ± 0.45	0.29 ± 0.022	12.7 ± 0.3	2.96 ± 0.55
LSTA	3.68 ± 0.03	8.93 ± 0.16	0.32 ± 0.041	13.1 ± 0.4	2.82 ± 0.47
LSTE	3.69 ± 0.02	8.54 ± 0.53	0.32 ± 0.055	13.1 ± 0.1	2.27 ± 0.54
HSCK	3.69 ± 0.01	7.63 ± 0.67	0.29 ± 0.032	13.1 ± 0.2	2.41 ± 0.83
HST0	3.66 ± 0.05	8.37 ± 0.23	0.33 ± 0.017	12.5 ± 1.1	2.91 ± 0.69
HSTA	3.66 ± 0.02	8.55 ± 0.38	0.34 ± 0.017	12.6 ± 0.6	2.21 ± 0.72
HSTE	3.69 ± 0.02	7.62 ± 0.67	0.29 ± 0.032	13.1 ± 0.2	2.41 ± 0.83

Abbreviation: LSCK, control wine with low sulfur; LST0, wine with low sulfur and addition of exogenous condensed tannins before fermentation; LSTA, wine with low sulfur and addition of exogenous condensed tannins in the rise period of acetaldehyde; HSCK, control wine with high sulfur; HST0, wine with high sulfur and addition of exogenous condensed tannins before fermentation; HSTA, wine with high sulfur and addition of exogenous condensed tannins in the rise period of acetaldehyde. Note: no remarkable differences among the wines (*p* > 0.05).

## Data Availability

The original contributions presented in this study are included in the article/Supplementary Materials. Further inquiries can be directed to the corresponding author(s).

## Supplementary Materials

The following supporting information can be downloaded at: https://www.mdpi.com/article/10.3390/molecules29225238/s1. Figure S1. HPLC chromatography of identified anthocyanins in Cabernet Sauvignon red wine. Abbreviations: 1: Malvidin 3-O glucoside; 2: Vitisin A; 3: Vitisin B; 4: Malvidin 3-O-(6-O-acetyl)-glucoside-pyruvic acid; 5: Malvidin 3-O-glucoside-ethyl-catechin (1); 6: Malvidin 3-O-(6-O-acetyl)-glucoside-acetaldehyde; 7: Malvidin 3-O-(6-O-acetyl)-glucoside; 8: Malvidin 3-O-(6-O-p-coumaryl)-glucoside-pyruvic acid; 9: Malvidin 3-O-(6-O-caffeoyl)-glucoside; 10: Malvidin 3-O-(6-O-trans-p-coumaryl)-glucoside.
