# Supplementary material for: Impact of Condensed Tannin and Sulfur Dioxide Addition on Acetaldehyde Accumulation and Anthocyanin Profile of Vitis vinifera L. Cv. Cabernet Sauvignon Wines During Alcoholic Fermentation
Source: Molecules. 2024 Nov 5;29(22):5238. doi: 10.3390/molecules29225238 (PMC11596415; doi:10.3390/molecules29225238)
Supplement: Supplementary file 1 [file molecules-29-05238-s001.zip › molecules-3259261-supplementary.pdf]

Impact of Condensed Tannin and Sulfur Dioxide Addition on Acetaldehyde Accumulation and Anthocyanin Profile of Vitis vinifera L. Cv. Cabernet Sauvignon Wines During Alcoholic Fermentation

Qinglong Wang [1], Xiaoqian Cui [1], Jiaqi Wang [1], Heqiang Chang [1], Junzhe Wang [1], Ang Zhang [2], Yang Zhou [1, 3], Zhiyong Xu [1, 3], Lingmin Dai [1,*] and Guomin Han [1,*]

1 School of Bioengineering, Qilu University of Technology (Shandong Academy of Sciences), Jinan 250353, China; wangqinglong975@163.com (Q.W.); 17863908085@163.com (X.C.); 19861406968@163.com (J.W.); 19862126289@163.com (H.C.); wjz001118@163.com (J.W.); zhywine@163.com (Y.Z.); xzy19890710@126.com (Z.X.)

2 Technology Center of Qinhuangdao Customs, Qinhuangdao 066004, China; zhanganggrape@hotmail.com

3 Wei Long Grape Wine Co., Ltd., Yantai 265704, China

* Correspondence: dailingmin@qlu.edu.cn (L.D.); gmhan@qlu.edu.cn (G.H.)

Figure S1. HPLC chromatography of identified anthocyanins in Cabernet Sauvignon red wine.

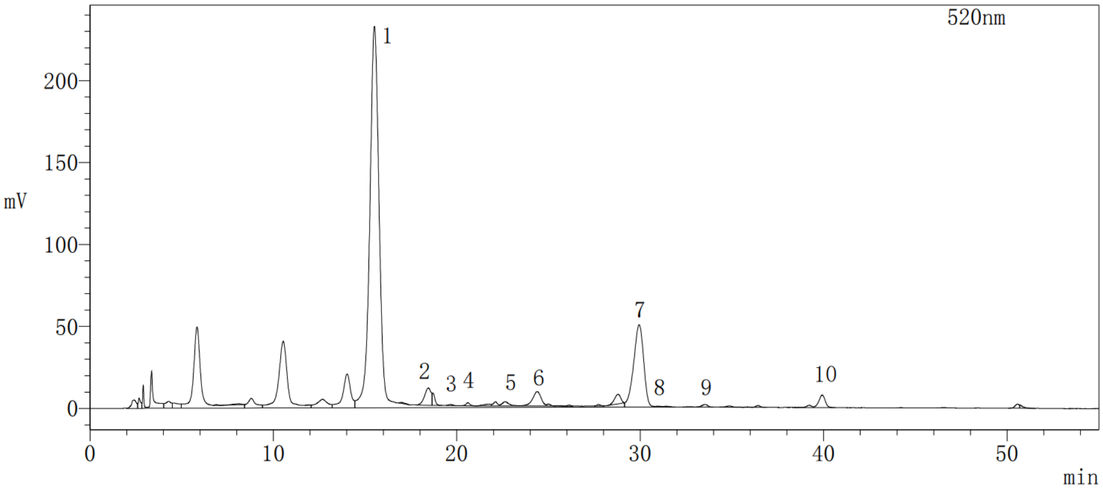

Figure S1. HPLC chromatography of identified anthocyanins in Cabernet Sauvignon red wine. Abbreviations: 1: Malvidin 3-O glucoside; 2: Vitisin A; 3: Vitisin B; 4: Malvidin 3-O-(6-O-acetyl)-glucoside-pyruvic acid; 5: Malvidin 3-O-glucoside-ethyl-catechin (1); 6: Malvidin 3-O-(6-O-acetyl)-glucoside-acetaldehyde; 7: Malvidin 3-O-(6-O-acetyl)-glucoside; 8: Malvidin 3-O-(6-O-p-coumaryl)-glucoside-pyruvic acid; 9: Malvidin 3-O-(6-O-caffeoyl)-glucoside; 10: Malvidin 3-O-(6-O-trans-p-coumaryl)-glucoside.
